# Discrete choice experiments: a primer for the communication researcher

**DOI:** 10.3389/fcomm.2025.1385422

**Published:** 2025-02-17

**Authors:** Reed M. Reynolds, Lucy Popova, Bo Yang, Jordan Louviere, James F. Thrasher

**Affiliations:** 1 Communication Department, University of Massachusetts, Boston, MA, United States; 2 School of Public Health, Georgia State University, Atlanta, GA, United States; 3 Department of Communication, University of Arizona, Tucson, AZ, United States; 4 University of South Australia, Adelaide, SA, Australia; 5 Department of Health Promotion, Education and Behavior, Arnold School of Public Health, University of South Carolina, Columbia, SC, United States

**Keywords:** discrete choice experiments, balanced incomplete block designs, fractional factorial designs, message evaluation tasks, conjoint analysis (CA)

## Abstract

Experiments are widely used in communication research to help establish cause and effect, however, studies published in communication journals rarely use discrete choice experiments (DCEs). DCEs have become a mainstay in fields such as behavioral economics, medicine, and public policy, and can be used to enhance research on the effects of message attributes across a wide range of domains and modalities. DCEs are powerful for disentangling the influence of many message attributes with modest sample sizes and participant burden. The benefits of DCEs result from multiple design elements including stimulus sets that elicit direct comparisons, blocked and/or fractional factorial structures, and a wide range of analytic options. Though sophisticated, the tools necessary to implement a DCE are freely available, and this article provides resources to communication scholars and practitioners seeking to add DCEs to their own methodological repertoire.

## Introduction

Imagine a researcher named Lauren wants to know how a person’s appearance contributes to first impressions. In many cultures, the face receives the most visual attention during initial encounters and it shapes inferences about personal characteristics (Gullberg and Holmqvist, 1999) in ways relevant for interpersonal relationships and social-influence campaigns alike (Moslehi et al., 2024), in terms of beauty, status, similarity, and so forth. As Lauren contemplates the topic, the abundance of influential factors becomes clear—face shape, facial expression, hair, eye color, etc. But with so many variables, she wonders how many experiments she needs to understand what really drives the process. As it turns out, the number may be smaller than most researchers realize. While the complexity of communication is increasingly studied (Ianovici et al., 2023; Sherry, 2015), conventional experimental designs in communication research limit the number of variables that can be manipulated within a single study either because of participant burden to respond to, or researcher burden to create large numbers of message conditions.

This article offers a primer on discrete choice experiments (DCEs; Carson and Louviere, 2011; Friedel et al., 2022), including tools and recommendations immediately usable by researchers. DCEs are an experimental paradigm underutilized in the field of communication (e.g., Cunningham et al., 2014; Iyengar and Hahn, 2009; Messing and Westwood, 2014), but prevalent in disciplines such as marketing (Carson et al., 1994; Louviere and Woodworth, 1983), healthcare (de Bekker-Grob et al., 2012; Folkvord et al., 2022; Lack et al., 2020; Quaife et al., 2018; Soekhai et al., 2019; Tünneßen et al., 2020), tobacco control (Regmi et al., 2018; Reynolds et al., 2022; Salloum et al., 2018; Thrasher et al., 2018b; Ntansah et al., 2025), policy impact assessment (Lagarde and Blaauw, 2009), and political science (Poertner, 2020). In contrast to traditional message-effects or message evaluation research, DCEs leverage comparison sets of multiple stimuli (often called choice sets), as well as blocked and/or fractional-factorial designs, and flexible analysis options that amplify statistical power to detect effects of message attributes. DCEs enable simultaneous testing of large numbers of independent variables without extremely large sample sizes and can be used in conjunction with standard survey items or other experimental inductions (Hawkins et al., 2014). The efficiency to estimate effects with small numbers of participants may be the clearest benefit of DCEs, but their flexibility also enables wide-ranging applications in message-evaluation, message-effects, and media-selection research. DCEs can quickly identify message features with the best chance to make an impact. Although DCEs are not applicable in every situation and are subject to several limitations, they merit additional attention by communication scholars.

### Overview of DCEs

DCEs build upon on foundations of general experimental design but may be unfamiliar even to experienced researchers. Experiments are indispensable for establishing cause and effect; they involve at least one induction (a.k.a. intervention, manipulation, treatment) that exposes subjects to contrasting conditions, with the goal of estimating an induction’s effect by comparing observations across those conditions (Memon et al., 2019). To accomplish this, experiments should ensure that all subjects or trials have equal probability of assignment to each condition (i.e., factor-level combination). Randomization allows this by preventing (on average) subject characteristics from correlating with condition assignment, which could bias estimates of induction effects. Experiments should also employ, to the extent possible, strict minimization of differences between conditions except for the focal variable targeted by the induction. If experimental groups differ in ways other than the intended treatment, the precise cause of differences in outcomes cannot be established because confounding factors might be responsible. Accordingly, experiments are most valuable when they can eliminate plausible alternative explanations for the observed effect.

An ideal experiment on communication effects would manipulate all relevant variables simultaneously using a full factorial design, however, large numbers of experimental factors are infeasible, and communication research typically includes only a small number of factors per experiment (e.g., Carpenter, 2013). This piecemeal approach is powerful if integrated into an ongoing research program, but can also be inefficient, requiring more subjects and more time overall. In addition, experimental conditions can be compared more meaningfully within a single study rather than across multiple studies because the benefits of randomization can be leveraged, giving each condition the same expected distribution for all individual differences. The family of DCE methods offers several benefits in this regard. To assist the presentation of terminology we provide a brief glossary of terms in Table 1.

Discrete choice experiments (DCE) refer to a collection of procedures, design characteristics, and analytic frameworks where participants compare and evaluate stimuli, usually presented in sets. Stimuli can take the form of messages or can depict profiles of entities or objects that each represent a unique combination of attributes (Lancsar and Louviere, 2008; Louviere et al., 2000). A DCE’s basic purpose is to infer the relative impact of each stimulus attribute on stimulus evaluation; in other words, to identify the message components responsible for perceptions of that message.^1^ In the context of communication research, stimuli may include most any kind of message and stimulus-features may include most any kind of message variable. Evaluations take the form of participant-provided comparisons or ratings of objects specified by the researcher. For example, our researcher, Lauren, may present sets of contrasting images of faces and ask subjects to select the one that appears most trustworthy. The task is simple, yet the design is distinct from conventional rating or selection tasks. Returning to our example, Lauren would have the ability to estimate the extent that perceptions of trustworthiness result from attributes of the eyes relative to the mouth expression, skin color, and so on. Although responses may occur at various levels of measurement, DCEs usually involve a ranking or choice task for each set, resulting in ordinal or dichotomous data (see Carson et al., 2022; Louviere et al., 2010). DCEs are related to the framework of conjoint analysis and stated preference designs (see Eggers et al., 2021; Louviere et al., 2010; Mühlbacher and Johnson, 2016). Below, we discuss specific design implementations.

#### Case 1 designs: attribute evaluation

**FIGURE 1:**
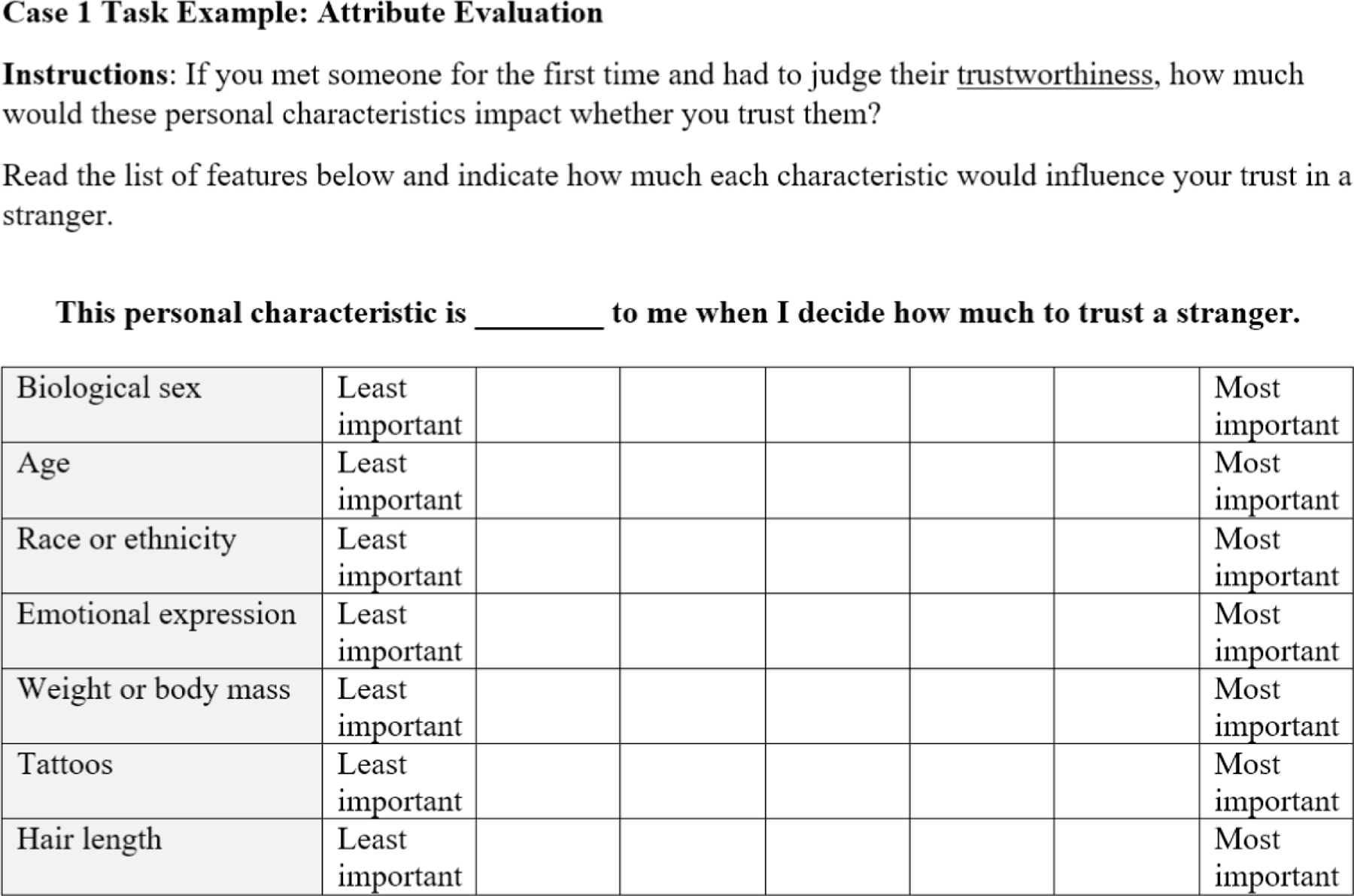
Example attribute evaluation task. Here, the evaluations are collected using semantic differential scales rather than dichotomous selection.

Because DCEs belong to a family of methods, we consider variations that serve a similar purpose but with perhaps more limitations than full-fledged DCEs. One such method is often called a case 1 design which elicits explicit attribute evaluations. Returning to our example case, Lauren could address trustworthiness inferences in a rudimentary way by giving subjects a written list of personal attributes and asking how important each feature is (perceived to be) for determining trustworthiness. Figure 1 illustrates a sample attribute evaluation (case 1) task that Lauren might use. It contains seven facial features identified as potentially relevant to trustworthiness evaluations. This task could be constructed as a simple selection of attributes with the most or least importance, however, ratings may also be used. In case 1 and case 2 designs, ratings may reduce estimation problems associated with dominant attributes (Soekhai et al., 2021).

Case 1 attribute evaluation designs have the advantage of simple construction and efficient implementation; however, they have limited ability to determine the actual effect of message features. If Lauren relied on this method, her study would have involved no actual facial displays, nor systematic variation to create levels of each attribute. From a design perspective, therefore, case 1 designs have limited ability to show the effects of said features. In addition, subjects are explicitly asked to predict the influence of each item, but predictions of this kind are susceptible to biases, as people often fail to realize or wish to conceal the cognitive processes underlying their decisions (Nisbett and Wilson, 1977). For an example of a case 1 design, see Cheung et al. (2016).

#### Case 2 designs: stimulus-attribute evaluation

**FIGURE 2:**
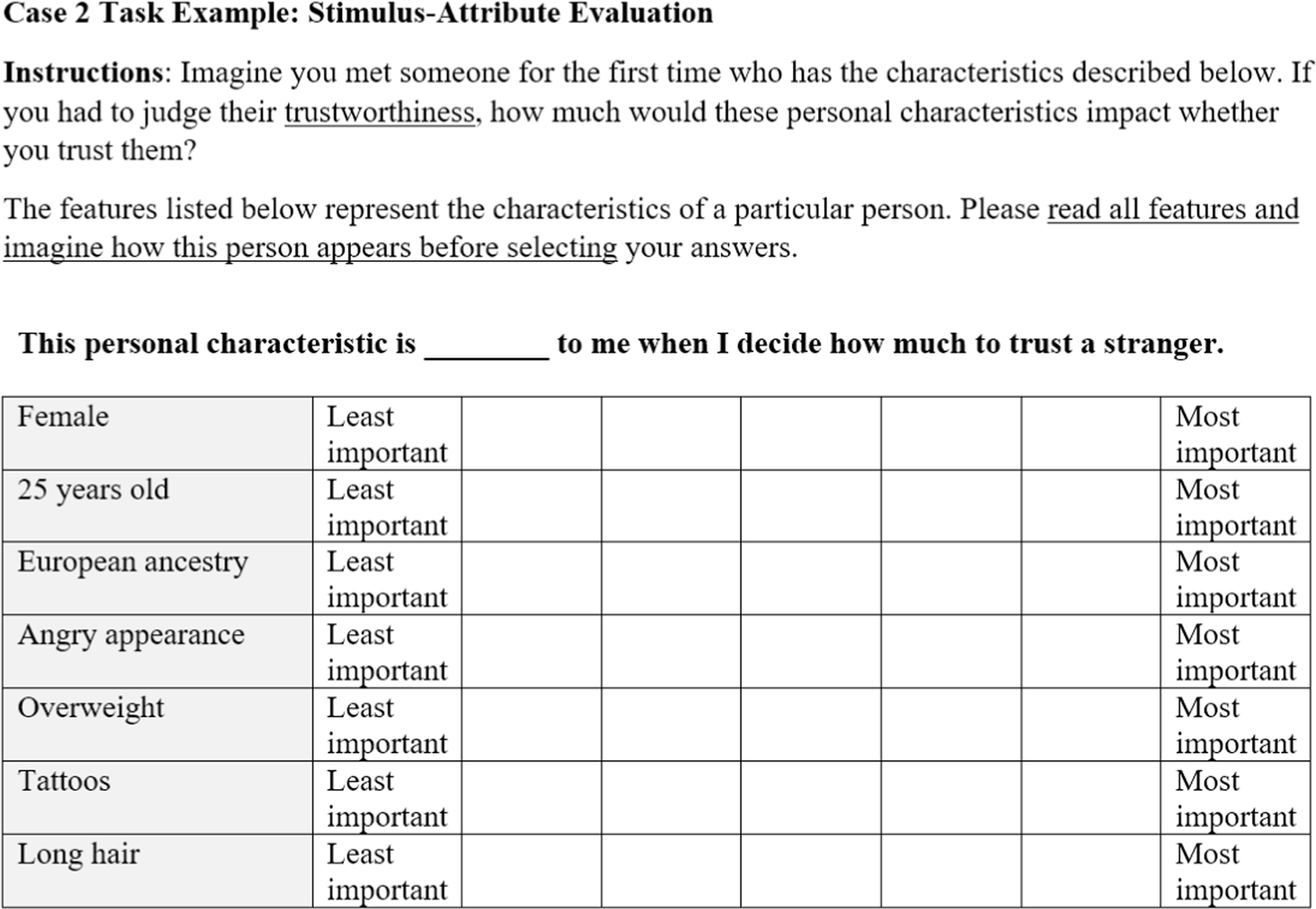
Example stimulus-attribute evaluation task. Here, the evaluations are collected using semantic differential scales rather than dichotomous selection.

Another method in the DCE family is the case 2, or *stimulus-attribute evaluation* design. As in the case 1 method described above, the researchers develop a list of features expected to influence evaluations of an object or message. In addition, researchers articulate levels of each feature category. In our example, the feature category “emotional expression” could be instantiated by the two levels, happy and angry. Levels can be constructed for every feature category of interest (e.g., eyebrow shape, skin tone), permitting a full or fractional factorial design. The method proceeds as a repeated measures design where articulated feature-level combinations are presented one-by-one. Most commonly, case 2 designs use stimuli formed by concrete descriptions of feature-level combination (e.g., Cheung et al., 2016), however, it is possible to use graphical representation as stimuli as well. The outcomes are participant evaluations of the importance or impact of specific features. As in case 1 designs, the researcher specifies the evaluative criterion, such as trustworthiness, attractiveness, competence, etc. Figure 2 illustrates a case 2 stimulus that Lauren might use in her study.

Case 2 designs have several advantages over case 1. Each attribute is tied to the particular level displayed, leaving less ambiguity about how participants interpret their meaning. Researchers also control the levels included or excluded from the study, based on relevance to the given research question. By using a factorial design that includes different combinations of attribute levels, researchers can also analyze nonlinear effects of each attribute type. For example, the description of the intensity of a smile could be manipulated by varying degrees, and the data could reveal that the apparent intensity of a smile has a curvilinear relationship with evaluations. The factorial design can also test interaction effects between attribute types; for example, in Lauren’s study on facial features, the data may reveal that individuals from out-groups are perceived as particularly untrustworthy when they are not smiling.

Although case 2 designs are more robust, they have several limitations. First, they rely on bias-prone introspection, like case 1 designs. Specifically, as shown in Figure 2, participants are asked to evaluate the impact of each attribute on their overall judgment of the stimulus. In other words, participants do not evaluate stimuli directly, rather, participants rate the impact of each attribute in shaping their evaluation. As a consequence, responses may be vulnerable to bias and a lack of ability to introspect about the causes of behavior and cognitive processes (Nisbett and Wilson, 1977). People may be influenced by perceived skin color, for instance, but fail to realize or admit the influence of that factor and therefore provide inaccurate responses. For examples of case 2 designs, see Cheung et al. (2016), Coast et al. (2006), and Soekhai et al. (2021).

Figure 2 shows another potential limitation of common case 2 designs. There, each attribute is instantiated as a specific level described in textual form. This is not inherently problematic, as messages often include textual elements. However, in this case the phenomenon of interest is a person’s visual appearance, and verbal descriptions (a) are subject to varied interpretations, and (b) place higher cognitive burden on participants to imagine the described attributes. This illustrates the importance of modality in conveying profile information and the benefits of stimuli that resemble the objects they represent.

#### Case 0 designs: stimulus evaluation

**FIGURE 3:**
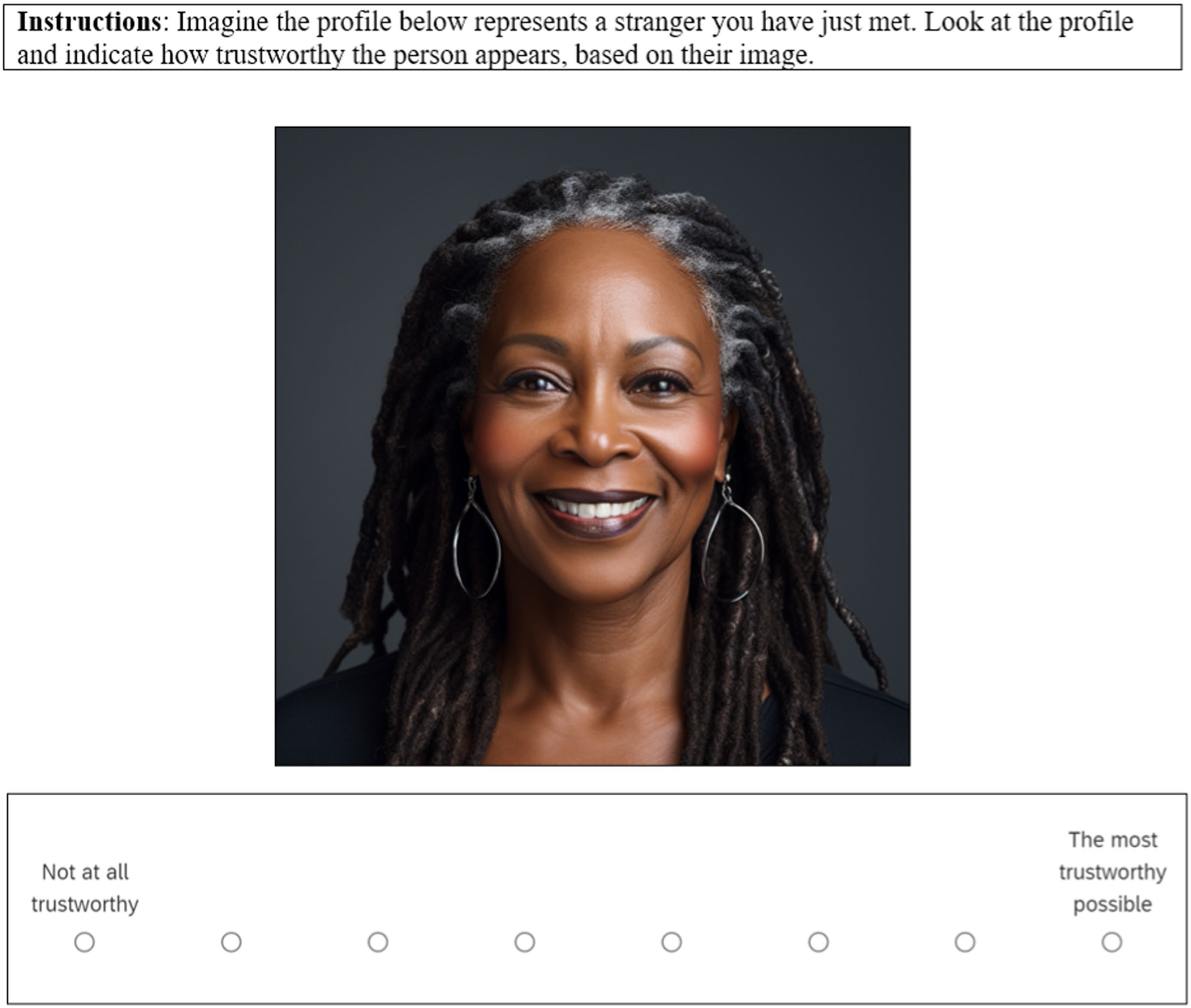
Example task from a stimulus evaluation design. Image generated with artificial intelligence software (Midjourney, 2024).

Although not generally considered a DCE, stimulus evaluation (SE) designs are an important point of comparison. These are the conventional designs commonly used in communication research (e.g., Bente et al., 2020; Reynolds et al., 2019), especially for message-effect studies. SE designs elicit evaluations of stimuli directly rather than evaluations of stimulus attributes. Just as case 2 DCEs, stimulus evaluation designs articulate all combinations of attribute-levels, but do elicit inferences about specific stimulus attributes. Typically, participants give separate evaluations of each stimulus, and researchers then estimate the effect of each attribute on subject evaluations, enabled by the factorial design (Judd et al., 2012). Figure 3 displays an example of a stimulus evaluation task for the trustworthiness study. Despite the merits of SE designs, DCEs are a more efficient alternative in many cases.

#### Case 3 designs: multi-stimulus discrete choice experiments

**FIGURE 4:**
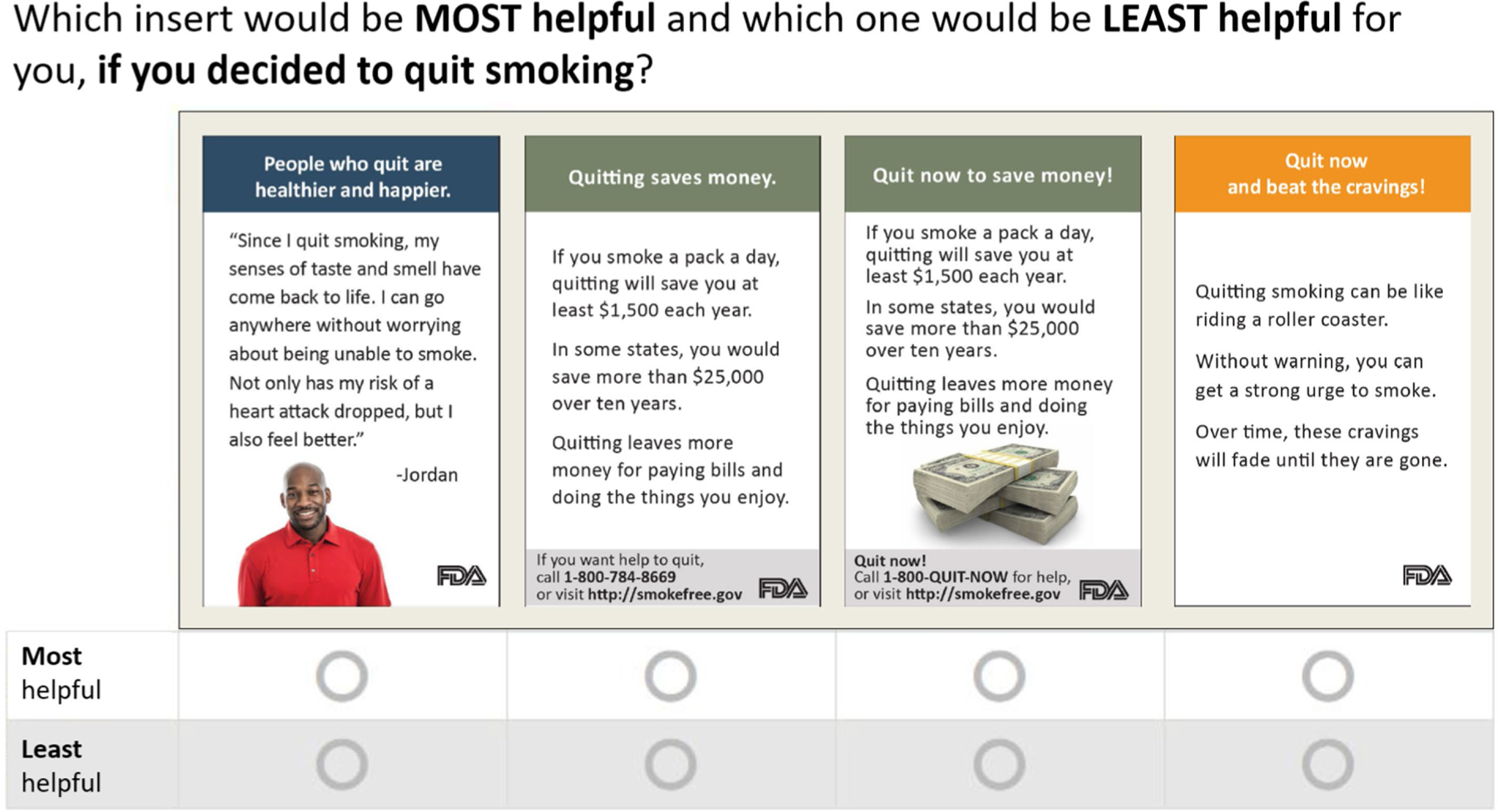
Example stimulus-comparison set (adapted from Thrasher et al., 2018a).

Below we present the commonly used and more sophisticated DCE designs that use some features of the designs previously discussed. DCEs use stimuli depicting attribute-level combinations to instantiate the range of relevant attributes. In addition, DCEs presenting multi-stimuli simultaneously, using sets to elicit comparative evaluations (Carson and Louviere, 2011). In DCEs, participants do not evaluate attributes or attribute levels,^2^ but directly evaluate the stimuli in each set, often by providing relative rankings of the options presented. For illustration, we have presented the work of Thrasher et al. (2018a) who developed sets of messages designed to motivate smokers to quit (see Figure 4). Messages varied along five attributes including message topic, information type (i.e., testimonial vs. factual), image (i.e., present vs. absent), call to action (i.e., present vs. absent), and contact information (i.e., present vs. absent). Although DCEs may be constructed with multiple evaluation tasks, here, participants selected the most and least helpful message out of each comparison set, applicable to the best-worst scaling analytic framework (discussed below). The design used by Thrasher et al. (2018a) had 64 possible message conditions, however, fractional factorial designs permit fewer messages and a manageable number of comparison sets (see below). DCEs go beyond traditional self-report techniques in several ways. They can more efficiently quantify the effect of stimuli, provide information about the relative importance of stimulus attributes to general audiences, and estimate each individuals’ sensitivity to a given attribute (Cleland et al., 2018; Turk et al., 2020). Comparison sets in DCE designs can include real or hypothetical stimuli (Cleland et al., 2018) and are applicable to virtually any context (Lancsar and Louviere, 2008).

The multi-stimulus design of DCEs has several advantages. First, the evaluation task can approximate real-life decision scenarios where individuals weigh trade-offs between competing options, consistent with Random Utility Theory (e.g., Gerasimou, 2010; Hess et al., 2018; Lancaster, 1966; Mas-Colell et al., 1995; McFadden, 1974; Thurstone, 1927). This can help maximize the ability to discern between even similar stimuli. In addition, multi-stimulus DCEs require no inference about which features are responsible for a given evaluation, allowing evaluations that generalize to real contexts (Cleland et al., 2018).

A number of DCE design elements require further consideration, including the selection and number of attributes, the number and size of comparison sets, blocked designs, fractional factorial designs, response measures, and analysis options. Below, we discuss these topics in detail and provide recommendations about the trade-offs implied by design choices. To summarize, Table 2 presents a concise overview of characteristics of each design.

### DCE design elements

#### Attributes and levels

DCEs begin like any experiment, with a clear research question and conceptualization of key variables. Although DCEs are flexible and efficient, judicious selection of factors and levels still helps satisfy limitations of sample size and participant attention. Once researchers have identified key attributes, they will determine the levels to include. Attribute levels should constitute meaningful categories that likely occur within the context under study. For Lauren’s study, she was aware of several cultural artifacts and stereotypes that influence rapport-building (Bente et al., 2020), leading to her decision to manipulate features that might be associated with stereotypes, such as ancestry or ethnicity, emotional expression, tattoos, etc. Ideally, chosen levels should span a wide range to capture the extremities of the attribute while maintaining realism. Intermediate levels may be important as well, especially where non-linear effects are suspected, but a weak induction (one with small differences between levels) can result in a failure to find an effect.

So-called “control” conditions may also be considered for each attribute, and researchers should consider what kind of reference category allows the most meaningful comparison. Critically, the goal is to eliminate confounding variables as plausible explanations for observed effects. Constructing control levels can be complex. For example, at times withholding content can serve as a control, whereas at other times filler content is more suitable to preserve realism and similarity in message length and task characteristics. When in doubt, a researcher can include multiple control conditions per attribute. In the context of facial-feature research, a control stimulus could depict a face with a neutral, or calm expression, rather than omitting features. Researchers should also consider their ability to produce the content required for each stimulus. Although constructing high-quality stimuli can be difficult and costly, researchers can also adapt content found in existing popular media or research literature.

When stimuli cannot be perfectly controlled, some attributes may vary in addition to the ones intended by the experimental induction. Although this could result in confounding, the problem can be addressed, to an extent, content coding stimuli. This means assigning additional attributes to stimuli and statistically accounting for their effect. This is critical when additional attributes are associated with experimental inductions and may be associated with the outcome of interest. For example, style of dress may be associated with the presence of tattoos, biasing estimates of tattoo effect. Appropriate coding and statistical adjustment may help prevent confounding, assuming the confounding variable is not perfectly correlated with the attribute of interest.

### Experimental design, stimulus construction, and comparison sets

DCE’s often use fractional factorial and balanced incomplete block designs (BIBDs) to accommodate the large number of possible attribute-level combinations represented by stimuli. Suppose that in Lauren’s study on facial feature effects, she decided to include seven factors. For the sake of simplicity, suppose she decided to have only two levels per factor (see Table 3). Multiplying the number of levels from each factor shows the design has 128 attribute-level combinations. This may be too many conditions to present to people during an experiment because of time, cost, participant willingness, or fatigue effects (Caussade et al., 2005). Large numbers of stimuli may also strain the researcher’s ability to generate the needed stimuli.

DCEs ultimately elicit evaluations within sets of contrasting stimuli, displaying two or more simultaneously, to estimate the influence of focal attributes. As indicated above, by stimulus we mean a concrete instantiation of a particular combination of message-attribute levels. Returning to the personal appearance context, Lauren could use cartoon illustrations, AI-generated images, or real photographs. The choice should consider the availability of existing content, the researcher’s own resources and skill at generating content representing the focal attributes, and the need to control for non-focal attributes.

**FIGURE 5:**
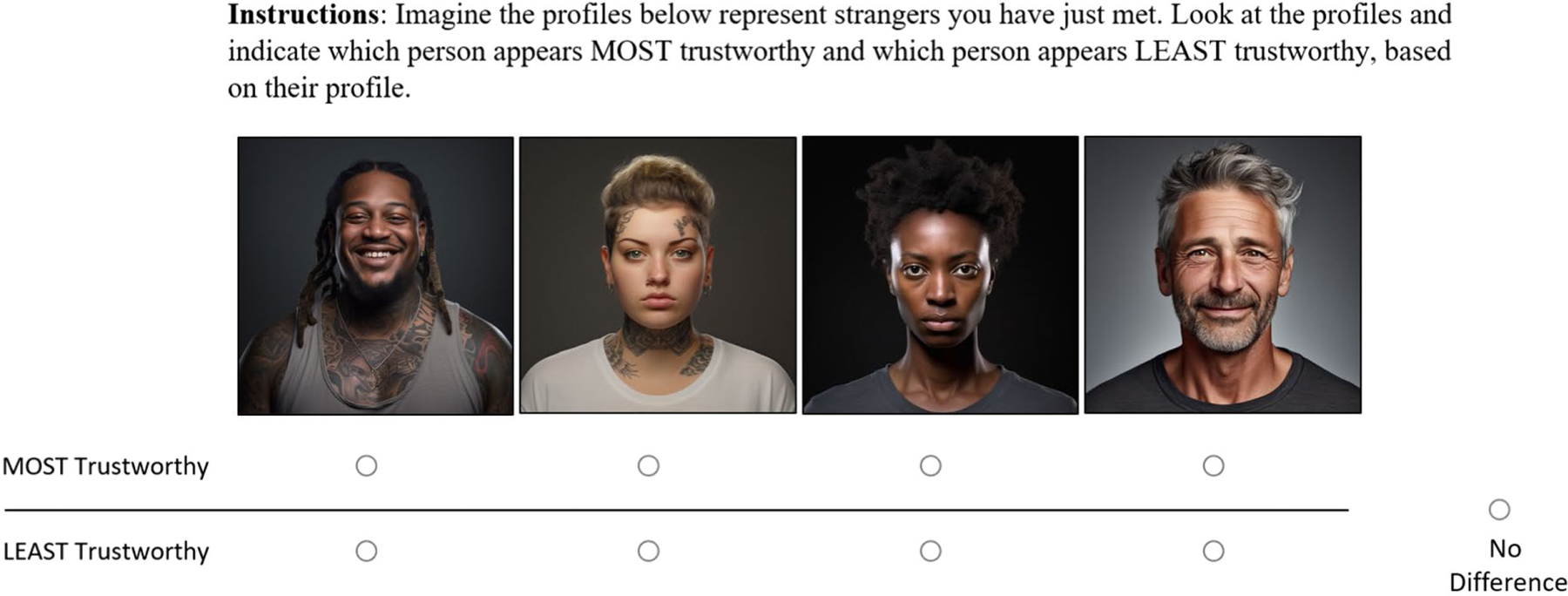
Example comparison set with stimuli representing combinations of features. Image generated with artificial intelligence software (Midjourney, 2024).

Figure 5 shows an example of four contrasting stimuli within a hypothetical comparison set. The first stimulus, for example, depicts a male of African ancestry with a happy expression, higher BMI, 25 years old, tattoos, and long hair. To estimate all main and interaction effects in this study, a full-factorial design with 128 stimuli would be needed. Following the recommendations of Reeves et al. (2016), researchers can also construct multiple stimuli per condition to reduce confounding of stimulus idiosyncrasies with attribute levels. This could be done, for example, by randomly sampling from pools of profiles or messages with multiple long hair styles and multiple short hair styles. This would help determine whether the long vs. short distinction is meaningful and potentially increase confidence about the generalizability of results.

### Balanced incomplete block designs

As the number of experimental conditions grows, due to more factors and/or more levels within factors, it may be impossible to expose each participant to all stimuli, even using comparison sets. In such cases, DCEs often adopt a balanced incomplete block design (BIBD) so that each stimulus is presented to a random subset of participants. The blocking procedure may help maintain efficient and unbiased estimation of attribute effects.

In a BIBD, stimuli are systematically assigned to blocks, or arrays, and participants are randomly assigned to receive the stimuli associated with a particular block. Within each block, stimuli are further assigned to comparison sets that facilitate the simultaneous display of multiple stimuli to a participant (discussed below under Comparison Set Construction). In some cases, stimuli may be repeated across blocks for the purpose of establishing common reference stimuli for all participants, potentially allowing greater statistical control of inter-rater differences.

Constructing blocks demands care. For example, if Lauren distributed stimuli so that only a single block contained happy expressions, the within-subject variance for that factor would be minimal, and it might then correlate with participant-level differences, given that randomization works imperfectly. To avoid this problem and estimate attribute effects more precisely, blocks are more effective when ‘balanced’, meaning they satisfy three conditions. First, blocks have equivalent size so that all participants receive the same number of stimuli (representations of attribute combinations). Second, stimuli are assigned such that each attribute level appears an equivalent number of times within each block. For example, Lauren would ensure that the number of stimuli with long hair is equivalent to the number with short hair within each block, and so on for each attribute. Third, each pair of attributes (i.e., each unique two-attribute combinations within a stimuli) appears an equal number of times in each block (Rink, 1987). Implementing BIBDs is complex but software can generate such designs for experiments with different numbers of factors and levels (e.g., the free R package DoE.base; Grömping, 2018).

To illustrate BIBDs, Table 4 presents an example block used by Lauren in her study. Notice that only 16 stimuli are included, requiring 1/8 of the possible 128. The specific stimuli included meet the requirements for a BIBD. The levels of each factor are displayed, to be represented numerically for the purpose of analysis (e.g., short hair = 0, long hair = 1). In this block, each attribute level occurs eight times and each pair of attribute-levels occurs four times. For example, stimuli 13–16 depict 55-year-old males, and no other stimuli have that combination. Analyzing the block also reveals that all factors are perfectly uncorrelated (*r* = 0), enhancing the efficiency of estimating independent effects. In a full factorial BIBD DCE, this block would be constructed alongside seven other 16-stimulus blocks to evenly distribute the other 112 stimuli. Appendix A illustrates sample code and output from the R package DoE.base that can help select a desired block design given a specified design.

### Fractional factorial designs

DCEs also commonly use fractional factorial designs (FFDs) to further reduce the number of stimuli required. Constructing fractional factorial designs uses the same criteria as BIBDs, however, some stimuli will be omitted from the design (not be included in any block). As with BIBDs, software tools exist to assist in generating these designs (e.g., DoE.base; Grömping, 2018). Choosing the attribute combinations to be omitted requires considering several assumptions and research objectives. Perhaps most important is the potential for non-additive effects among the factors; from our example study, this could occur if happy expressions influence trustworthiness differently on account of another facial feature. Fractional factorial designs can be specified to allow estimation of some or none of the possible interaction effects. In the DCE literature, the concept *resolution* captures the extent to which experimental effects are confounded within a given FFD design. Put simply, higher resolution designs involve less confounding between and among main and interaction effects. Box and Hunter (1961) discuss the concept in detail and define three of the most common categories of fractional factorial designs. As they state, a resolution 3 design confounds main effects with two-factor interactions. A resolution 4 design does not confound main effects with two-factor interactions, but two-factor interactions are confounded with one another. In resolution 5 designs, “no main effect or two-factor interaction is confounded with any other main effect or two-factor interaction, but two factor interactions are confounded with three factor interactions” (Box and Hunter, 1961, p. 319).

Higher resolution designs involve less confounding but generally require more stimuli. Designs of resolution less-than 3 are not useful because they confound main effects with other main effects. Importantly, only a full factorial design can estimate all main and interaction effects; thus, fractional factorial designs will fail to observe interaction effects and they will produce biased estimates of main effects if particular interactions do exist. Therefore, we recommend that fractional factorial designs be used with caution and in a way that main effects are unconfounded with at least all two-way interactions (i.e., resolution IV design or higher). Even without *a priori* expectations of interaction effects, prudence should require evidence of no interaction before proceeding with an FFD. The risk of bias is real because interaction effects are commonplace in communication research (e.g., Keller and Lehmann, 2008; Lang and Yegiyan, 2008; Reynolds, 2020). Moreover, the ability to test for interactions is a strength of multi-factor experiments that may be missed when omitting conditions. Appendix B displays example R code and output to help select a suitable fractional factorial balanced incomplete block design.

### Random stimulus sampling

As an alternative to blocked designs, perhaps the simplest way to accommodate excessively large numbers of attribute combinations is through random sampling of stimuli. In Lauren’s study, a unique random subset of the 128 stimuli could be selected for each participant. Randomization requires no complex blocking procedure and does not omit any portion of the factorial space. In this way it is less likely to produce biased estimates of main effects that result from confounding with interactions. Randomization is also easy to implement at the point of survey construction if the set of all stimuli can be generated. Recent research has also shown that random stimulus-sampling designs do not lose much efficiency as compared with blocked designs, especially when population parameters are uncertain (Walker et al., 2018).^3^ Despite the advantages of random stimulus sampling, like the full factorial design, they may not be feasible if the entire set of possible stimuli cannot be constructed, for example, if it is too expensive to do so. As another limitation, simple random stimulus sampling does not ensure that each participant is exposed to equal (or any) instances of each attribute level. In aggregate this is not problematic because general attribute effects can still be estimated, however, if one is interested in modelling individuals (e.g., Louviere, 2013) then a blocked design may be preferable to ensure that sufficient attribute combinations are presented to every participant (e.g., see Das et al., 2018).

### Comparison set construction

In many non-DCE designs, stimuli are presented one-by-one (i.e., the size of each set is 1). In contrast, DCEs typically present multiple stimuli simultaneously. In this way, evaluation occurs with direct comparisons between stimuli within the set. By using a comparison task, a single set can generate information about multiple stimuli. Comparison sets will contain at least two stimuli but most often contain four to efficiently generate evaluations for subsequent analysis (Caussade et al., 2005). As the number of stimuli within each comparison set grows, the difficulty of comparing stimuli tends to increase, particularly when stimuli reflect many complex attributes (DeShazo and Fermo, 2002). Developing comparison sets involves deciding how many stimuli will appear within each set, and then deciding how many times stimuli will reappear across sets. The total number of comparison sets must accommodate these parameters. Repeating stimuli across comparison sets generates more comparisons, allowing more precise estimates of attribute effects. Importantly, pretesting may be necessary to assess task difficulty and participant fatigue. Table 5 displays an example collection of comparison sets for Lauren’s 16-stimulus FFD.

Assigning stimuli to comparison sets generally applies the same criteria used for BIBDs. First, any particular comparison set should not contain multiple instances of the same stimulus, as that would involve comparing a stimulus to itself. Second, the property of balance can enhance parameter estimate precision. Specifically, all stimuli can appear an equal number of times across comparison sets. For example, each stimulus in Table 5 appears five times. The headings Stimulus A-D indicate the unique stimuli to be displayed for each comparison set. During implementation, stimuli can be displayed in various spatial orientations (e.g., horizontally or vertically), and the position of stimuli within comparison sets can be randomized and recorded if order effects are a concern. A third criterion for balanced sets involves each *pair* of stimuli (i.e., each unique 2-stimulus combination) occurring an equal number of times across comparison sets. Table 5 illustrates this, as each stimulus co-occurs with every other stimulus exactly once. When these design criteria are met, stimuli should receive the same number of evaluations and estimates of stimulus-attribute effects should be more precise. Note that balanced comparison sets are only possible for some combinations of design parameters. Designs will be more efficient if they approximate balance, even if perfect balance cannot be achieved. Alternately, researchers may use random sampling of stimuli (without replacement) to create comparison sets. Random sampling may be less efficient but remains unbiased (Walker et al., 2018). It is less efficient because it does not guarantee maximum contrast between attributes within comparison sets, but it is unbiased because randomization removes, on average, any association between profile evaluation and other variables of interest.

### Length considerations for DCEs

Researchers should consider the acceptable testing burden when setting design parameters such as the number of attributes, attribute levels, factorial structure, number of blocks, size of choice sets, number of choice sets, and type of evaluation tasks. Although the issue of maximum length remains controversial (Hess et al., 2012), there is some evidence that error variance and participant attrition increase as the number of comparison sets approaches 20 or more, perhaps due to fatigue (Bech et al., 2011); however, this reduction of power does not imply that parameter estimates will be biased (Louviere, 2004). Error variance may also be inflated in initial DCE tasks within an experiment (Louviere, 2013), suggesting that a training set may be helpful. The influence of DCE length, including the set number and set size, depends on factors including participant motivation, processing ability, and testing modality (Savage and Waldman, 2008). A researcher can empirically assess the effect of fatigue by estimating differences (e.g., in means, variances, or covariances) associated with presentation order. According to a recent meta-analysis on DCEs in the domain of tobacco control, the number of comparison sets ranged from 4 to 24 (*M* = 10.4, *SD* = 5.9; Regmi et al., 2018). For more on BIB designs, see Louviere et al. (2015) and Van der Linden et al. (2004).

#### Measurement scales and DCE tasks

DCEs are designed to elicit evaluations of stimuli or the effect stimuli are perceived to have. Conceptually, by evaluation we mean a person’s ascription of a quality to the object being evaluated. Decisions about the task and instrumentation can influence results enormously (Reynolds, 2020). Researchers should decide which message quality or perceived message effect they would like to address with their DCE, and this becomes the evaluative criterion given to participants. Lauren’s example study focuses on evaluations of trustworthiness—how much a person would trust another based on facial appearance. Lauren could as easily implement another evaluative criterion, such as attractiveness, friendliness, or similarity. In fact, DCE protocols can include multiple evaluations of the same comparison sets (e.g., trustworthiness and attractiveness). Pretesting is advisable to ensure that the evaluation task is clear, distinct, and within participants’ ability to perform.

One of the most prevalent DCE evaluation tasks relies on comparative judgments within each comparison set. Known as “best-worst” scaling, this method asks subjects to identify (a) the stimulus that best exemplifies the evaluative criterion, and (b) the one that worst exemplifies the evaluative criterion (Marley and Louviere, 2005). A best-worst task is less cognitively demanding than ranking every stimulus within a set, although it implies a full ranking when comparison sets contain only 3 stimuli. To collapse best-worst choices into a single indicator, Louviere et al. (2015) describe a method of recoding the data as 1 (best), −1 (worst), or 0 [not selected; for examples of best-worst scaling, see Najafzadeh et al. (2012) and Wright et al. (2017)]. Figure 5 displays an example of best-worst scaling applied to Lauren’s facial-feature study. Best-worst scaling is most often used with comparison sets of size 3 or 4. Best-worst scaling does have important limitations. For example, as an ordinal scale, it does not indicate the cardinal value of the evaluations provided; it will be unclear whether the option selected as “best” is considered good or bad. In addition, best-worst scaling may not capture the magnitude of differences between stimuli. For example, in a forced choice, similar stimuli will be given a rank that may reflect only a small difference.

Researchers have developed several ways to address these limitations of best-worst evaluations. For example, evaluation tasks can include a “no difference” option alongside stimuli, or researchers may add the question as a follow-up to every evaluation task. This inclusion informs researchers about whether participants truly differentiated the stimuli within a given comparison set. Comparison sets where “no difference” is indicated can be excluded from analysis, or models can be compared with and without these data. If excluding data, a researcher may want to determine whether any variables of interest are associated with the “no difference” selection. Empirical evidence shows that DCEs and best-worst scaling in DCEs can provide accurate estimates of the relative impact of stimulus attributes on individual evaluations, such as consumer preferences (Louviere et al., 2015; Salampessy et al., 2015). DCE’s can include multiple evaluative tasks simultaneously to achieve the most robust findings. For example, Lauren might ask which profiles seem more or less trustworthy than the average person, or simply elicit quantitative evaluations (e.g., ratings) from which rankings can be inferred.

### DCE data and analysis

DCEs can estimate the relative effect of each stimulus attribute on stimulus evaluations. They can also account for other variables such as individual participant-level differences, other between- or within-subject experimental conditions, or other stimulus characteristics (including additional evaluations elicited concurrently). Raw data may resemble those displayed in Table 6, that were simulated to resemble Lauren’s facial feature experiment. The data represent the responses of one participant across all comparison sets. Numbers in the two right-most columns indicate which of the 16 stimuli was selected as appearing most and least trustworthy for each comparison set.

For analysis, this raw data can be combined with those from Tables 3, 4 and restructured to enable the appropriate regression model. In particular, the attribute-level indicators should be included. A long-format data structure can be created with stimulus-presentations as the unit of analysis, expanding the number of observations. A stimulus presentation is the unique instance that each stimulus was presented to a particular person. In Lauren’s study (per Table 4), each stimulus is presented five times per subject. Therefore, the long-form data should have a number of cases equal to the number of stimuli times the number of presentations for each stimulus times the number of raters. Table 7 displays example data for Lauren’s facial feature study, showing rows from the first five comparison sets for the first participant. The BWS column is a best-worst-score described by Louviere et al. (2015) that (in this case) combines the most-trustworthy and least-trustworthy items into a single variable. The most and least choices can be analyzed in separate models, which allows estimation of asymmetry between choosing vs. rejecting (Krucien et al., 2019; Shafir, 1993). Some attributes may have a bigger impact on being rated worst (least) relative to being rated best (most). There may be cases where best-choice and worst-choice models indicate effects of opposite direction for the same attribute. For example, attributes that generate ambivalent responses from individuals, or polarizing evaluations across subpopulations may be more likely to be rated best *and* worst.

With the data structured this way, several analytic techniques are available, depending on the research questions and viability of model assumptions. A simple approach could use one of the dichotomous outcome variables (Most or Least) and estimate a variety of logistic regression models (Long and Freese, 2006; Sadique et al., 2013). This approach overcomes some limitations of the linear probability model applied to a dichotomous outcome, such as out-of-bounds predictions and heteroscedasticity (but see Hellevik, 2009).

As usual with DCEs, the data here are clustered in several respects; this means that rows do not represent independent observations. For example, within a choice set presentation, the likelihood of selecting a stimulus depends on the likelihood of selecting the alternatives. Also, evaluations made by the same participant are likely to have similar characteristics, relative to evaluations from others. In addition, design elements may cause clustering due to effects of blocks, presentation order, etc. (Louviere et al., 2008). Clustering causes additional variance in evaluations that may be of interest or may be considered a nuisance (Cameron and Miller, 2015; Galbraith et al., 2010; McNeish and Kelley, 2019). Analysis of DCE data should account for clustered data where applicable, as failing to do so may bias parameter estimates and produce inaccurate standard errors. Several approaches are available, each with advantages and disadvantages (Galbraith et al., 2010). Specifically, researchers may aggregate observations within clusters to generate non-clustered data. Researchers may also estimate fixed-effect models to statistically adjust for variables responsible for the clustering (Huang, 2016). Another approach is to estimate a mixed-effect models. A mixed-effect model includes random-effect components that allow parameter estimates—such as means or regression coefficients—to vary across clusters (Hole and Kolstad, 2012; Hossain et al., 2018; Sándor and Wedel, 2002). Mixed-effect models can quantify how much variability exists between and within clusters, for example, how much message features influence people in different ways.

Multiple tools are available to analyze clustered DCE data. For example, Stata’s CM module and its cmmixlogit command can perform the analysis to include both fixed and random effects. Selecting the appropriate model also can be empirically guided. For example, using likelihood-ratio tests, one can compare the fit of competing models (e.g., Lewis et al., 2011; Norris et al., 2006), such as fixed vs. random effects models. Here, overfitting is a concern (Lever et al., 2016), and in general simpler models are preferable unless their fit is substantially worse, or they are theoretically infeasible.

Regardless of the specific model used, researchers should be mindful of the multiple factors that influence evaluations. These commonly include variance associated with differences between comparison sets (and the different alternatives) and differences between participants (for discussion of additional sources of variance, see Hess and Rose, 2012). Accounting for these factors enables more accurate estimates of the general effect of each attribute. Both wide and long-form data permit several types of co-predictors. For example, the researchers can model the effect of participant-level characteristics such as age, sex, extraversion, and so forth, including interactions between such characteristics and stimulus attributes (e.g., Kim and Park, 2017; King et al., 2007). The models can include additional evaluations, for example, ratings of the profile’s attractiveness as a correlate of trustworthiness. Models can also include other experimental factors such as distractor conditions.

As with typical regression models, interaction terms can be added to estimate non-additive effects; however, as discussed earlier, fractional factorial designs compromise the ability to test all possible interactions between attribute categories. Attribute-by-attribute interactions must be pre-specified and be reflected in the design if a FFD is used. In Lauren’s study, she might test what stimulus attributes promote or diminish perceptions of trustworthiness, what groups of participants are more sensitive to a given cue, and what situations are more or less likely to bias trustworthiness attributions (e.g., after priming or persuasive messages). Recent scholarship has also developed frameworks for modelling single individuals from repeated-measures DCEs, including person-specific cue-sensitivity, variability, as well as latent cluster analysis (Louviere, 2013; Frischknecht et al., 2014). Data can also be expanded into a so-called “exploded” form (Chapman and Staelin, 1982; Lancsar et al., 2017), generated by inferring new observations from the choices actually observed, relying on the Independence of Irrelevant Alternatives (IIA) assumption (see Buckell et al., 2018; Herne, 1997).

While the long-form data give the researcher flexibility, the data can be aggregated for more basic analysis. For example, Louviere et al. (2015) discusses one method of averaging across raters. After this transformation the data have a number of rows equal to the number of total stimulus-presentations across all stimuli. In this formulation, a conditional logit model can be used to estimate the impact of attributes on the aggregated choices. One limitation of this approach is the inability to estimate effects resulting from participant-level variables. Ultimately, researchers should decide what assumptions are reasonable for their design, and what techniques are most applicable to their research goals. Many assumptions can be empirically tested with model diagnostics.

### Effect sizes for DCEs

Effect sizes convey the magnitude of association among variables and can be estimated in numerous ways (Ferguson, 2016). As presented below in our simulated data analysis, some DCE effects can be represented as differences in means or proportions, bivariate association, or regression coefficients. A researcher should consider their research objectives when choosing effect size statistics. Some effect size metrics lead to substantively different interpretations (McGrath and Meyer, 2006), while others are simply linear transformations that are more or less familiar to different audiences. Importantly, effect sizes will depend on each variable’s designated levels and observed variance, as well as model specification. For our simulated DCE, the effect of hair length is a contrast between long and short within the context of the study, and not a universal effect of hair length.

Regression coeffects are most common in DCE analysis, but can be easily mis-interpreted. They estimate independent effects, adjusting for other stimulus-level and participant-level variables. Odds ratios (ORs) have been among the most commonly reported effect sizes in DCEs (de Bekker-Grob et al., 2012) because of the limitations of OLS for categorical or ordinal outcomes (but see Hellevik, 2009). ORs are a standard output of statistical software and represent the change in odds of an outcome associated with per-unit changes or category comparisons in the predictor variable (for formulas, see Schechtman, 2002). Odds ratios can be difficult to compare, however, because their values neither linearly nor monotonically convey strength of association. In addition, as a type of unstandardized, unnormalized coefficient, odds ratios are influenced by the scale of variable increments as well as variance. Several normalization procedures are available for DCE coefficients that can help gauge and compare effect sizes (Gonzalez, 2019; Lancsar et al., 2007). One approach represents effect sizes as the impact relative to other model predictors, for example, as a pairwise ratio, or as a proportion of the model’s overall predictive power. Regardless of the approach, scale-normalization or standardization are critical for comparing coefficients within a study.

### Power analysis for DCEs

Power analysis can estimate the likelihood that a design will detect an effect of a given size, or determine what design is necessary to achieve a particular power. Failing to consider the power of a DCE may lead to uninformative results. Recently, de Bekker-Grob et al. (2012) conducted a review of power analysis practices for DCEs. As they discuss, when determining sample size for DCEs, key inputs are (1) desired significance level, (2) desired power, (3) intended statistical model to be used (e.g., multinomial logit, rank-order logit), (4) anticipated effect sizes, and (5) design characteristics including the number of parameters to be estimated, the number of stimuli per comparison set, and the number of comparison sets to be used. de Bekker-Grob et al. (2012) include R code that calculates the sample size needed from the parameters listed above. In addition, they provide simplified but commonly-used formula to roughly estimate a *minimum* number of subjects needed for DCEs. They provide the formula as *N* > 500*c* / (*t* · *a*), where *t* is the number of comparison sets assigned to each participant, *a* is the number of alternative within each set, and *c* is the product of the number of levels for the two largest factors (de Bekker-Grob et al., 2012).

### Example DCE analysis

Data were simulated to illustrate results that might be obtained from Lauren’s DCE on personal attributes and perceived trustworthiness. In this case, long-form non-exploded data were used due to their analytic flexibility and to reduce sensitivity to violations of the IIA assumption. Initially, descriptive statistics were calculated to show the proportion of selections rated most and least trustworthy for each level of the seven factors (see Table 8). Expected proportions (chance-level) are 0.25 because participants selected one stimulus out of four for each task. Results show some factors deviate from chance-level proportions. Regression analysis will examine these associations in more detail.

Using the simulated data, mixed-effect logistic regression was conducted with the best (most) and worst (least) as dependent variables in separate models. This tests the symmetry between coefficients of best and worst models, an assumption of combining best and worst choices into a single scale. As displayed in Table 9, each attribute (F1-F7) was entered as a fixed-effect predictor, along with indicators for the fixed-effect of each comparison set and the left-to-right position of each stimulus within each comparison set. Although fixed-effect models can account for clustered data structures (Huang, 2016), this becomes less feasible with larger numbers of clusters. Here, for example, choices are clustered within participants, but including a fixed effect would require 600 participant-indicator variables, a computationally demanding process that also reduces the ratio of observations per parameter estimate to unacceptable levels (see Vittinghoff and McCulloch, 2007). For illustration, participants were specified as a random factor with random intercepts. In addition, cluster-robust standard errors were calculated to adjust for clustering within participants and because the participant is presumed to be the basic sampling unit in this example.

Results of the simulated data in Table 9 show the overall model is significant. Coefficients are reported two ways. First, odds ratios (OR) represent the model-adjusted independent effect of each predictor on the odds of a stimulus being selected relative to not being selected (see Schechtman, 2002). For ORs, coefficients from 1 to ∞ indicate greater likelihood, and coefficients from 1 to 0 indicate reduced likelihood. Second, relative impact weights (RIW) quantify the magnitude of effect, normalizing the scaling factor of each variable, expressed as a percentage of the other predictors displayed (Gonzalez, 2019).

Results of the simulated data show that several stimulus attributes were associated with stimulus evaluations. In addition, results indicate asymmetry between most and least models. Specifically, in the most-trustworthy model, European ancestry (versus African) was associated with a 1.41 times increased likelihood of being evaluated as *most* trustworthy (relative to not being selected), *p* < 0.001. This result would be consistent with the presence of racial stereotypes that influence interpersonal perception. If results were symmetrical between most and least models, European ancestry would be associated with a reduced likelihood of being selected *least* trustworthy, however, there was no effect in the least-trustworthy model. A Brant test (Liu, 2009) formally tested the hypothesis of symmetry between all coefficients, indicating significant asymmetry, χ^2^(7) = 44.7, *p* < 0.001. If desired, variables can be omitted to test asymmetry for specific coefficients. As a result, in this case most and least choices should not be combined (see Krucien et al., 2019). The moderating effect of DCE tasks (e.g., most vs. least choices) may be of interest, potentially indicating framing effects, ambivalence, or heterogenous effects across sub-populations. Here, we have focused on the most-trustworthy model; in general, positively valenced choices generally show less error variance (Krucien et al., 2019).

Model 1 also shows a significant effect of emotional expression and tattoos; angry expressions were associated with a 0.81 reduction in odds of being evaluated as *most* trustworthy, relative to happy expressions, adjusting for other stimulus attributes, *p* < 0.001. Similarly, tattoos were associated with 0.54 times decreased odds of being evaluated as *most* trustworthy, *p* > 0.001.

Model 3 shows a significant interaction between participant sex and emotion within the stimulus, OR = 1.59, *p* < 0.001. That pattern of results indicates that, compared to male participants, female participants were significantly less likely to evaluate a profile as most trustworthy when they expressed anger, controlling for other stimulus attributes. Participant sex also moderated the effect of stimulus ancestry on trustworthiness, OR = 0.58, such that women were significantly more likely than men to rate people as most trustworthy if they are European. These kinds of results could indicate differences in stereotypes across different populations.^4^ In all models, the effect of tattoos was the strongest, indicated by its relatively high RIW.

Note that the coefficient for participant sex is not displayed. In strictly comparative discrete choice models, participant-level direct effects are not meaningful without respect to the attributes of the stimuli being evaluated. In addition, Table 9 shows zero variance in intercepts between participants for the random effect. This is ensured by the design, as the comparative task involved the same number of sets and selections for all individuals. An alternative level of measurement, such as quantitative rating scales would allow variance in intercepts between participants; it would also enable direct effects of participant characteristics to be estimated, rather than only interactions between stimulus attributes and participant characteristics.

As a reminder, the design in this example has a resolution 3 fractional factorial structure meaning it cannot estimate interactions between stimulus attributes. It may be, for example, that attributes like tattoos and expression have non-additive effects. As discussed previously, estimating these would require a higher resolution design (resolution 4 or higher, or a full factorial design).

Distributions of individual-level coefficients can also be estimated. Here, the correlation between each two-level factor and each selection (most—not most) was estimated for each participant (Rodgers and Nicewander, 1988). As a participant-level variable, the correlation magnitude can then be correlated with other variable or treated as an outcome. Table 10 shows such output, including confidence intervals, and differences in correlations between men and women. Importantly, when treating the participant as the unit of analysis, using one observation per participant is generally appropriate, or adjusting standard errors to avoid inflating Type 1 error. In total, the simulated results illustrate how DCEs can reveal factors contributing to interpersonal perceptions of trustworthiness. This approach can be leveraged for other kinds of message effects or message selection studies.

### Limitations of DCEs

The limitations of DCEs inform their suitability for a given research context. First, because they can accommodate designs with many experimental conditions, DCEs may be time consuming or expensive to implement. DCE designs also entail more complexity than conventional designs, although this obstacle can be minimized with freely available software and experience. In addition, DCEs are not equally suitable for all types of stimuli. For example, DCEs may induce high cognitive burden when messages are difficult, complex, or require a long processing times (Bryan and Dolan, 2004). In such cases, participants may have less ability to successfully compare stimuli within sets, or may fatigue quickly after a few sets (but see the above discussion on fatigue effects). DCE researchers can compensate by selecting designs with fewer stimuli within sets and fewer sets overall.

Because they emphasize comparisons between groups of stimuli, DCEs are not suitable for assessing all types of message effects. This is likely true where the intended effect requires a single message exposure without contrasting content, or highly immersive long-form audio-visual material. Additional research is needed to test the boundaries of DCE’s applicability to various kinds of message-effects research. As discussed above, the comparative tasks of typical DCEs provide estimates of the relative impact of message attributes rather than the absolute evaluation of messages. The relative impact of attributes may be most helpful to target factors for use in subsequent research or content generation. This limitation can be removed by including other measures within or alongside a DCE design.

## Conclusion

DCEs have become a mainstay in several fields and have been used to predict critical real-world outcomes as well as to test theory. However, communication scholars have yet to take full advantage of their potential. As we have demonstrated in this article, DCE’s are highly applicable to studies on the effects of message attributes across a wide range of domains and modalities. Efficiency is perhaps their main benefit, as DCEs can disentangle the influence of many attributes with modest sample sizes and reasonably short experimental sessions. The benefits of DCEs accrue as a result of multiple design elements, including the use of stimulus sets to elicit direct comparisons, blocked or fractional factorial structures, and the breadth of analytic frameworks available. Though sophisticated, the tools necessary to implement a DCE are freely available, and this article provides resources to communication researchers who examine large numbers of factors at once and who seek to implement DCEs themselves.

## Supplementary Material

Supplementary Table 2

Supplementary Table 1

The Supplementary material for this article can be found online at: https://www.frontiersin.org/articles/10.3389/fcomm.2025.1385422/full#supplementary-material

## Figures and Tables

**TABLE 1:** Brief glossary of DCE terms.

Term	Definition
Attribute/feature	A discernable characteristic of a stimulus, either subject to experimental variation or content coding within DCEs. For example, the attribute of message source can be varied to reflect different media organizations.
Balanced incomplete block design (BIBD)	A design where stimuli are systematically assigned to blocks such that blocks have an equivalent number of stimuli, each attribute level appears an equivalent number of times within each block, and each pair of attributes appears an equal number of times in each block. Respondents are then assigned to receive the stimuli associated with particular block(s).
Best-worst scaling	An evaluation task where respondents identify the stimulus that best exemplifies the evaluative criterion (e.g., attractiveness, trustworthiness, etc.), and the stimulus that least exemplifies the evaluative criterion
Block	A design element containing a subset of stimuli to which participants can be assigned to evaluate that particular subset
Comparison set	A group of stimuli presented simultaneously to a respondent along with an evaluation task
DCE (discrete choice experiment)	A method where participants evaluate or select stimuli with experimentally varied attributes, presented in comparison sets, with the purpose of (a) estimating effects of stimulus attributes on participant evaluations, (b) differentiating between stimulus tendencies to elicit particular evaluations, and/or (c) differentiating between participant sensitivities to particular stimulus attributes
Efficiency (of designs)	The amount of resources in respondents, stimuli, and/or observations required by a design to estimate an effect with a given level of precision
Evaluation	Respondent-provided classifications, comparisons, or ratings of stimuli according to a criterion
Factor	A variable that represents systematic differences across experimental conditions
Factor (between-subjects)	An experimental factor for which a single condition is assigned per respondent, varying across (between) respondents but remaining constant within respondents
Factor (within-subjects)	An experimental factor for which multiple conditions are assigned per respondent
Fractional factorial design	A multiple-factor experiment where observations are obtained for only some factor-level combinations, usually selected systematically
Full factorial design	A multiple-factor experiment where observations are obtained for each factor-level combination
Induction/manipulation	A protocol that systematically exposes subjects to contrasting conditions defined by the researchers
Odds ratio	The change in odds of an outcome associated with per-unit changes or category comparisons in the predictor variable
Profile	A type of stimulus that represents an object or entity
Relative impact weight	An effect size normalized by variable scale and expressed as a proportion relative to one or more other model predictors
Resolution	The degree to which experimental effects (main or interaction) are confounded within a given fractional factorial design. Higher values indicate less confounding
Stimulus	A perceptible object or representation of an object or entity. Stimuli may take the form of messages, profiles, or other audio-visual presentations
Stimulus presentation	Each unique instance that a given stimulus is presented to a particular person within a study

**TABLE 2:** Summary table of design characteristics.

Design type	Initial steps	Benefits	Limitations	Common analysis options	Example studies
Case1: Attribute Evaluation	• Identify most important message attribute-types for research question • Define evaluative criterion and rating/comparison task • No need to articulate concrete levels for each attribute-type	• Simple to construct • Requires little participant time to complete • Ideal for initial data collection	• Participants may interpret attribute descriptions differently • Possibly subject to biased introspection (i.e., discrepancy between perceived effect and true effect) • Poorly equipped to assess interaction effects or non-linear associations	• Mean comparisons (e.g., average differences between attribute ratings/choices) • Associations between attribute ratings/choices (e.g., correlation, factor analysis) • Participant-level predictors of attribute ratings or choices (e.g., regression)	• Cheung et al. (2016) • Webb et al. (2021) • Louviere et al. (2015)
Case 2: Stimulus-Attribute Evaluation	• Identify most important message attribute-types for research question • Articulate each level of each attribute-type • Define evaluative criterion and rating/comparison task • Generate fractional factorial design (although full factorial is optional) • Generate profiles describing unique combinations of features	• Moderately simple to construct • Less ambiguity about attribute levels (than case 1 designs) • May only require textual descriptions of stimulus attributes (rather than full stimulus construction) • Can test interaction effects and non-linear associations	• Possibly subject to biased introspection	• Mean comparisons (e.g., differences between attribute ratings/choices) • Linear models to estimate attribute-by-attribute main and interaction effects • Estimate non-linear attribute effects • Estimate espondent-level predictors of attribute ratings/choices	• Cheung et al. (2016) • Coast et al. (2006) • Louviere et al. (2015) • Soekhai et al. (2019)
Stimulus Evaluation Design	• Identify most important message attribute-types for research question • Articulate each level of each attribute-type • Define evaluative criterion and rating/comparison task • Generate stimuli representing unique combinations of features	• Requires no introspection about effects of particular features • Evaluation tasks are simple, with low testing burden	• Requires large investment in stimulus construction • Participation may be lengthy with numerous stimuli	• Attribute-level predictors of stimulus ratings/ choices, including attribute-by-attribute interaction effects (e.g., regression, mixed-effect models) • Participant-level moderators of attribute-level effects on ratings/choices (e.g., mixed-effect models, multi-level models)	• Bente et al. (2020) • Reynolds et al. (2019) • Visch et al. (2014)
Case 3: Multi-Stimulus DCE	• Identify most important message attribute-types for research question • Articulate each level of each attribute-type • Define evaluative criterion and rating/comparison task • Generate fractional factorial design (although full factorial is optional) • Generate stimuli representing unique combinations of features • Generate stimulus sets for comparative evaluation	• High efficiency for eliciting message evaluations • Does not require introspection about the effects of each attribute • Can test interaction effects • Does not require construction of all stimuli • Many analysis options	• Requires careful consideration of design • Requires pretesting evaluation or rating task • Participants may need a training set	• Attribute-level predictors of stimulus ratings/choices, including attribute-by-attribute interaction effects (e.g., conditional logit regression, mixed-effect models) • Participant-level moderators of attribute-level effects on ratings/choices (e.g., mixed-effect models, multi-level models)	• Bansback et al. (2012) • Kim and Park (2017) • Rubin et al. (2006) • Shang et al. (2018) • Thrasher et al. (2018a,b)

**TABLE 3:** Example stimulus factors and levels for DCE.

Factor	(1)	(2)	(3)	(4)	(5)	(6)	(7)
Level	Sex	Age	Ancestry	Expression	BMI	Tattoos	Hair
0	Female	25	African	Happy	20	No	Short
1	Male	55	European	Angry	30	Yes	Long

**TABLE 4:** Example balanced incomplete block for 2×2×2×2×2×2×2 design.

	Attribute						
Stimulus	Sex	Age	Ancestry	Expression	BMI	Tattoos	Hair
1	Female	25	African	Happy	20	No	Short
2	Female	25	African	Angry	30	Yes	Short
3	Female	25	European	Happy	30	No	Long
4	Female	25	European	Angry	20	Yes	Long
5	Female	55	African	Happy	30	Yes	Long
6	Female	55	African	Angry	20	No	Long
7	Female	55	European	Happy	20	Yes	Short
8	Female	55	European	Angry	30	No	Short
9	Male	25	African	Happy	20	No	Short
10	Male	25	African	Angry	30	Yes	Short
11	Male	25	European	Happy	30	No	Long
12	Male	25	European	Angry	20	Yes	Long
13	Male	55	African	Happy	30	Yes	Long
14	Male	55	African	Angry	20	No	Long
15	Male	55	European	Happy	20	Yes	Short
16	Male	55	European	Angry	30	No	Short

**TABLE 5:** Example block of comparison sets for a multi-profile DCE.

Comparison set	Stimulus A	Stimulus B	Stimulus C	Stimulus D
1	2	5	8	14
2	1	5	6	7
3	5	9	12	16
4	4	11	5	15
5	3	5	10	13
6	1	3	2	4
7	2	6	9	11
8	7	16	13	2
9	10	2	15	12
10	1	8	9	10
11	6	8	13	15
12	4	7	8	12
13	3	8	11	16
14	14	1	15	16
15	3	14	12	6
16	7	10	11	14
17	14	9	13	4
18	13	11	12	1
19	10	16	4	6
20	9	7	3	15

This design is configured with 16 stimuli in total, and 4 stimuli per comparison set. Stimuli labels A-D represent the display position within each set. Stimulus numbers represent those shown in Table 3.

**TABLE 6:** Example raw data for DCE with best-worst evaluative task.

Participant	Comparison set	Most trustworthy	Least trustworthy
1	1	8	2
1	2	6	1
1	3	12	16
1	4	5	4
1	5	5	10
1	6	2	3
1	7	9	6
1	8	16	2
1	9	15	2
1	10	10	8
1	11	13	6
1	12	12	7
1	13	11	8
1	14	16	14
1	15	12	6
1	16	10	14
1	17	13	14
1	18	12	1
1	19	10	6
1	20	15	3

“Most trustworthy” and “Least trustworthy” indicate which stimulus was selected for the respective task.

**TABLE 7:** Example long-form data for DCE with best-worst evaluative task.

Participant	Set	Stimulus	Most	Least	BWS	Sex	Age	Ancestry	Expression	BMI	Tattoos	Hair
1	1	8	1	0	1	0	1	1	1	1	0	1
1	1	14	0	0	0	1	1	0	1	0	0	0
1	1	2	0	1	−1	0	0	0	1	1	1	1
1	1	5	0	0	0	0	1	0	0	1	1	0
1	2	5	0	0	0	0	1	0	0	1	1	0
1	2	1	0	1	−1	0	0	0	0	0	0	1
1	2	7	0	0	0	0	1	1	0	0	1	1
1	2	6	1	0	1	0	1	0	1	0	0	0
1	3	12	1	0	1	1	0	1	1	0	1	0
1	3	9	0	0	0	1	0	0	0	0	0	1
1	3	16	0	1	−1	1	1	1	1	1	0	1
1	3	5	0	0	0	0	1	0	0	1	1	0
1	4	15	0	0	0	1	1	1	0	0	1	1
1	4	5	1	0	1	0	1	0	0	1	1	0
1	4	11	0	0	0	1	0	1	0	1	0	0
1	4	4	0	1	-1	0	0	1	1	0	1	0
1	5	5	1	0	1	0	1	0	0	1	1	0
1	5	3	0	0	0	0	0	1	0	1	0	0
1	5	10	0	1	-1	1	0	0	1	1	1	1
1	5	13	0	0	0	1	1	0	0	1	1	0

BWS refers to best-worst scaling.

**TABLE 8:** Proportion of selections by condition.

	Proportions		
Factor	Selected most trustworthy	Selected least trustworthy	Chance-level occurrence
Sex			
Female	0.249	0.251	0.250
Male	0.252	0.249	0.250
Age			
25	0.254	0.251	0.250
55	0.246	0.249	0.250
Ancestry			
African	0.224	0.247	0.250
European	0.277	0.253	0.250
Expression			
Happy	0.266	0.232	0.250
Angry	0.234	0.269	0.250
BMI			
20	0.244	0.249	0.250
30	0.256	0.251	0.250
Tattoos			
No	0.297	0.218	0.250
Yes	0.203	0.282	0.250
Hair			
Short	0.249	0.246	0.250
Long	0.252	0.254	0.250

n(choices) = 48,000. all factors intercorrelate at *r* = 0.

**TABLE 9:** Predictors of perceived trustworthiness.

		Attribute effect estimates [99% CI]					
		Model 1 most trustworthy		Model 2 least trustworthy		Model 3 most trustworthy	
Predictor		OR	RIW	OR	RIW	OR	RIW
Stimulus sex		1.02 [0.95, 1.09]	1.32	0.99 [0.92, 1.06]	1.34	0.99 [0.91, 1.08]	0.94
0 = female; 1 = male							
Stimulus age		0.96 [0.90, 1.03]	3.27	1.00 [0.93, 1.07]	0.06	1.00 [0.91, 1.09]	2.16
0 = 25; 1 = 55							
Stimulus ancestry		1.41 [1.30, 1.52]*	26.39	1.04 [0.97, 1.12]	5.42	1.84 [1.68, 2.01]*	17.26
0 = African; 1 = European							
Stimulus expression		0.81 [0.75, 0.87]*	16.51	1.28 [1.19, 1.37]*	31.01	0.64 [0.58, 0.71]*	10.70
0 = happy; 1 = angry							
Stimulus BMI		1.04 [0.97, 1.12]	3.32	1.01 [0.94, 1.09]	1.51	1.00 [0.91, 1.09]	2.20
0 = 20; 1 = 30							
Stimulus tattoos		0.54 [0.50, 0.57]*	47.91	1.53 [1.42, 1.65]*	53.75	0.53 [0.48, 0.57]*	32.53
0 = no; 1 = yes							
Stimulus hair		1.02 [0.94, 1.10]	1.29	1.06 [0.99, 1.13]	6.91	0.99 [0.90, 1.08]	0.53
0 = short; 1 = long							
Interactions							
P. sex by S. sex						1.06 [0.95, 1.19]	1.61
P. sex by S. age						0.92 [0.82, 1.02]	2.20
P. sex by S. ancestry						0.58 [0.52, 0.65]*	13.95
P. sex by S. expression						1.59 [1.42, 1.79]*	11.98
P. sex by S. BMI						1.10 [0.98, 1.23]	2.41
P. sex by S. tattoos						1.02 [0.91, 1.14]	0.49
P. sex by S. hair						1.04 [0.92, 1.17]	1.02
Random intercept (participant)							
σ2		0.00	0.00			0.00	
ICC (participant)		0.00	0.00			0.00	
Model *p*-value (wald *χ*2)		<0.001	<0.001		<0.001		
Sample Size							
Participants	600		600		600		
Comparison sets	12,000		12,000		12,000		
Choices	48,000		48,000		48,000		

Model 1 = most-trustworthy choice was dependent variable, Model 2 = least-trustworthy choice was dependent variable, Model 3 is identical with Model 1 except interaction terms have been added. OR = Odds ratio. RIW = relative impact weight. Mixed-effect logistic regression was used with random intercepts for Participants. *p < 0.01. P. sex = participant sex (0 = female, 1 = male). Fixed effects for choice-set and profile position are not displayed.

**TABLE 10:** Correlations between stimulus attributes and most-trustworthy selections by participant sex.

	Mean correlation [99% confidence interval]		
Stimulus attribute	All participants (*n* = 600)	Female participants (*n* = 314)	Male participants (*n* = 286)
Sex	0.00 [−0.01, 0.02]	−0.00 [−0.02, 0.01]	0.01 [−0.01, 0.03]
Age	−0.01 [−0.02, 0.00]	−0.01 [−0.02, 0.01]	−0.01 [−0.03, 0.03]
Ancestry	0.06 [0.13, 0.07]	0.11 [0.10, 0.13]	0.00 [−0.01, 0.02]
Expression	−0.04 [−0.05, −0.02]	−0.08 [−0.10, −0.06]	0.01 [−0.00, 0.03]
BMI	0.01 [0.00, 0.03]	0.02 [−0.00, 0.03]	0.02 [0.00, 0.03]
Tattoos	−0.11 [−0.12, −0.10]	−0.11 [−0.12, −0.09]	−0.11 [−0.12, −0.09]
Hair length	0.00 [−0.01, 0.02]	0.00 [−0.02, 0.02]	0.01 [−0.01, 0.03]

## Data Availability

The original contributions presented in the study are included in the article/Supplementary material, further inquiries can be directed to the corresponding author.

## Footnotes

1 The method does not rule out simultaneously estimating effects of participant characteristics or other factors. In other words, DCEs can synergize with conventional survey and experimental paradigms.

2 Although multiple methods may be used in a single experiment.

3 As Walker et al. (2018) show, the most substantial way to increase design efficiency is by excluding “dominant” or “dominated” stimuli. In economic contexts these can be clearly identified as some attribute levels are universally preferred over others (e.g., lower prices). There is less universality in communication contexts, however, and so pruning stimuli from the design space *a priori* may be inadvisable.

4 But note that these data were simulated using random parameters and variable labels for illustration.
