# Supplementary material for: Discrete choice experiments: a primer for the communication researcher
Source: Front Commun (Lausanne). Author manuscript; Available in PMC 2025 Aug 21. (PMC12366800; doi:10.3389/fcomm.2025.1385422)
Supplement: Supplementary Table 2 [file NIHMS2092451-supplement-Supplementary_Table_2.docx]

**APPENDIX B**

**Example DoE.base Code and Output:**

**Balanced Incomplete Blocks (Orthogonal Arrays)**

**Code**


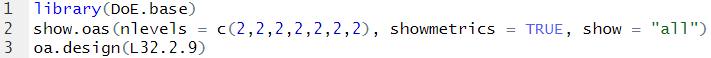


**Explanation**

Here, line 1 loads the R package (must already be installed). Line 2 runs command *show.oas* that shows the possible orthogonal arrays (BIBs) for the specified design. The design is specified with the *nlevels* argument, input as a vector with an integer representing the number of levels of each factor. Here we specified a 2x2x2x2x2x2x2 design. The output identified over 1,400 BIBs of resolution 3 or higher. These form the basis of fractional factorial designs and represent combinations of attributes such that all factors are uncorrelated. Supplemental Table 2 shows the first 130 BIBs provides by *DoE.base* for this design, sorted in order of descending resolution (degree of effect confounding). Supplemental Table 2 also displays the block size, or number of stimuli for each block. After selecting a BIB, the attribute-level combinations may be generated with the block name using the command in line 3.

Line three generates a matrix specifying the attribute-level combinations associated with each stimulus for the block named *L32.2.9*, that has a resolution of at least 4, and a block size of 32. Note that some BIBs have a block size larger than the full factorial.

| **Supplemental Table 2.** Partial list of BIBs for Example (2^7^) Fractional Factorial Design. | | | |
| --- | --- | --- | --- |
| Block Number | Block Name | Block Size | Resolution |
| 1 | L96.2.7.3.1 | 96 | 5.592 |
| 2 | L256.2.19 | 256 | 5 |
| 3 | L256.2.19 | 256 | 5 |
| 4 | L32.2.9 | 32 | 4.5 |
| 5 | L64.2.7.8.1 | 64 | 4.5 |
| 6 | L72.2.12.3.2 | 72 | 4.333 |
| 7 | L48.2.9.3.1 | 48 | 4.293 |
| 8 | L48.2.7.6.1 | 48 | 4.184 |
| 9 | L32.2.16 | 32 | 4 |
| 10 | L64.2.12.4.2 | 64 | 4 |
| 11 | L64.2.8.4.3 | 64 | 4 |
| 12 | L80.2.12.5.1 | 80 | 4 |
| 13 | L96.2.20.4.2 | 96 | 4 |
| 14 | L128.2.28.4.2 | 128 | 4 |
| 15 | L128.2.20.4.3 | 128 | 4 |
| 16 | L128.2.15.8.1 | 128 | 4 |
| 17 | L128.2.8.8.2 | 128 | 4 |
| 18 | L192.2.36.4.3 | 192 | 4 |
| 19 | L256.2.52.4.3 | 256 | 4 |
| 20 | L256.2.24.8.2 | 256 | 4 |
