# Supplementary material for: Discrete choice experiments: a primer for the communication researcher
Source: Front Commun (Lausanne). Author manuscript; Available in PMC 2025 Aug 21. (PMC12366800; doi:10.3389/fcomm.2025.1385422)
Supplement: Supplementary Table 1 [file NIHMS2092451-supplement-Supplementary_Table_1.docx]

**APPENDIX A**

**Example DoE.base Code and Output:**

**Full Factorial Blocked Design**

**Code**


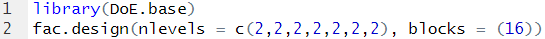


**Explanation**

Here, Line 1 loads the R package (must already be installed). Line two executes command *fac.design*, generating a matrix representing a full-factorial design (displayed below in Supplemental Table 1). The design is specified with the *nlevels* argument, input as a vector with an integer representing the number of levels of each factor. Here we specified a 2x2x2x2x2x2x2 design. The blocks argument specifies how many blocks to generate, with stimuli evenly distributed across blocks. Each block will be, to the extent possible, a balanced incomplete block. Specifically, each attribute-level will appear the same number of times within each block (and across blocks), each attribute-level pair will occur the same number of times, and attributes will be uncorrelated. Note that such a design is not possible for all numbers of blocks, given the number of stimuli specified by the design.

**Supplemental Table 1**. Example Full Factorial Balanced Incomplete Block Design.

| Prof. Num. | Block Num. | Attrib. 1 | Attrib. 2 | Attrib. 3 | Attrib. 4 | Attrib. 5 | Attrib. 6 | Attrib. 7 |
| --- | --- | --- | --- | --- | --- | --- | --- | --- |
| 1 | 1 | 0 | 1 | 0 | 0 | 1 | 0 | 1 |
| 2 | 1 | 0 | 1 | 0 | 1 | 0 | 1 | 0 |
| 3 | 1 | 0 | 0 | 1 | 0 | 1 | 1 | 0 |
| 4 | 1 | 1 | 0 | 0 | 1 | 1 | 0 | 0 |
| 5 | 1 | 1 | 1 | 1 | 1 | 1 | 1 | 1 |
| 6 | 1 | 1 | 1 | 1 | 0 | 0 | 0 | 0 |
| 7 | 1 | 1 | 0 | 0 | 0 | 0 | 1 | 1 |
| 8 | 1 | 0 | 0 | 1 | 1 | 0 | 0 | 1 |
| 9 | 2 | 1 | 1 | 1 | 0 | 0 | 0 | 1 |
| 10 | 2 | 1 | 0 | 0 | 0 | 0 | 1 | 0 |
| 11 | 2 | 0 | 1 | 0 | 0 | 1 | 0 | 0 |
| 12 | 2 | 1 | 1 | 1 | 1 | 1 | 1 | 0 |
| 13 | 2 | 0 | 1 | 0 | 1 | 0 | 1 | 1 |
| 14 | 2 | 1 | 0 | 0 | 1 | 1 | 0 | 1 |
| 15 | 2 | 0 | 0 | 1 | 0 | 1 | 1 | 1 |
| 16 | 2 | 0 | 0 | 1 | 1 | 0 | 0 | 0 |
| 17 | 3 | 0 | 0 | 1 | 0 | 1 | 0 | 0 |
| 18 | 3 | 1 | 1 | 1 | 1 | 1 | 0 | 1 |
| 19 | 3 | 0 | 1 | 0 | 0 | 1 | 1 | 1 |
| 20 | 3 | 1 | 1 | 1 | 0 | 0 | 1 | 0 |
| 21 | 3 | 0 | 0 | 1 | 1 | 0 | 1 | 1 |
| 22 | 3 | 0 | 1 | 0 | 1 | 0 | 0 | 0 |
| 23 | 3 | 1 | 0 | 0 | 0 | 0 | 0 | 1 |
| 24 | 3 | 1 | 0 | 0 | 1 | 1 | 1 | 0 |
| 25 | 4 | 1 | 1 | 1 | 0 | 0 | 1 | 1 |
| 26 | 4 | 1 | 0 | 0 | 1 | 1 | 1 | 1 |
| 27 | 4 | 0 | 1 | 0 | 1 | 0 | 0 | 1 |
| 28 | 4 | 0 | 1 | 0 | 0 | 1 | 1 | 0 |
| 29 | 4 | 1 | 1 | 1 | 1 | 1 | 0 | 0 |
| 30 | 4 | 0 | 0 | 1 | 0 | 1 | 0 | 1 |
| 31 | 4 | 1 | 0 | 0 | 0 | 0 | 0 | 0 |
| 32 | 4 | 0 | 0 | 1 | 1 | 0 | 1 | 0 |
| 33 | 5 | 0 | 0 | 1 | 1 | 1 | 0 | 1 |
| 34 | 5 | 0 | 0 | 1 | 0 | 0 | 1 | 0 |
| 35 | 5 | 1 | 0 | 0 | 0 | 1 | 1 | 1 |
| 36 | 5 | 1 | 1 | 1 | 0 | 1 | 0 | 0 |
| 37 | 5 | 0 | 1 | 0 | 0 | 0 | 0 | 1 |
| 38 | 5 | 1 | 0 | 0 | 1 | 0 | 0 | 0 |
| 39 | 5 | 0 | 1 | 0 | 1 | 1 | 1 | 0 |
| 40 | 5 | 1 | 1 | 1 | 1 | 0 | 1 | 1 |
| 41 | 6 | 1 | 0 | 0 | 0 | 1 | 1 | 0 |
| 42 | 6 | 0 | 0 | 1 | 0 | 0 | 1 | 1 |
| 43 | 6 | 1 | 1 | 1 | 0 | 1 | 0 | 1 |
| 44 | 6 | 0 | 1 | 0 | 1 | 1 | 1 | 1 |
| 45 | 6 | 1 | 1 | 1 | 1 | 0 | 1 | 0 |
| 46 | 6 | 1 | 0 | 0 | 1 | 0 | 0 | 1 |
| 47 | 6 | 0 | 0 | 1 | 1 | 1 | 0 | 0 |
| 48 | 6 | 0 | 1 | 0 | 0 | 0 | 0 | 0 |
| 49 | 7 | 1 | 1 | 1 | 0 | 1 | 1 | 0 |
| 50 | 7 | 0 | 0 | 1 | 0 | 0 | 0 | 0 |
| 51 | 7 | 1 | 0 | 0 | 0 | 1 | 0 | 1 |
| 52 | 7 | 0 | 1 | 0 | 0 | 0 | 1 | 1 |
| 53 | 7 | 0 | 1 | 0 | 1 | 1 | 0 | 0 |
| 54 | 7 | 1 | 1 | 1 | 1 | 0 | 0 | 1 |
| 55 | 7 | 0 | 0 | 1 | 1 | 1 | 1 | 1 |
| 56 | 7 | 1 | 0 | 0 | 1 | 0 | 1 | 0 |
| 57 | 8 | 1 | 0 | 0 | 1 | 0 | 1 | 1 |
| 58 | 8 | 0 | 1 | 0 | 1 | 1 | 0 | 1 |
| 59 | 8 | 0 | 0 | 1 | 0 | 0 | 0 | 1 |
| 60 | 8 | 1 | 1 | 1 | 1 | 0 | 0 | 0 |
| 61 | 8 | 1 | 1 | 1 | 0 | 1 | 1 | 1 |
| 62 | 8 | 1 | 0 | 0 | 0 | 1 | 0 | 0 |
| 63 | 8 | 0 | 1 | 0 | 0 | 0 | 1 | 0 |
| 64 | 8 | 0 | 0 | 1 | 1 | 1 | 1 | 0 |
| 65 | 9 | 1 | 1 | 0 | 0 | 0 | 0 | 0 |
| 66 | 9 | 0 | 0 | 0 | 0 | 1 | 1 | 0 |
| 67 | 9 | 0 | 0 | 0 | 1 | 0 | 0 | 1 |
| 68 | 9 | 1 | 0 | 1 | 1 | 1 | 0 | 0 |
| 69 | 9 | 0 | 1 | 1 | 1 | 0 | 1 | 0 |
| 70 | 9 | 1 | 0 | 1 | 0 | 0 | 1 | 1 |
| 71 | 9 | 0 | 1 | 1 | 0 | 1 | 0 | 1 |
| 72 | 9 | 1 | 1 | 0 | 1 | 1 | 1 | 1 |
| 73 | 10 | 0 | 1 | 1 | 0 | 1 | 0 | 0 |
| 74 | 10 | 1 | 1 | 0 | 1 | 1 | 1 | 0 |
| 75 | 10 | 1 | 0 | 1 | 1 | 1 | 0 | 1 |
| 76 | 10 | 0 | 0 | 0 | 0 | 1 | 1 | 1 |
| 77 | 10 | 1 | 1 | 0 | 0 | 0 | 0 | 1 |
| 78 | 10 | 0 | 1 | 1 | 1 | 0 | 1 | 1 |
| 79 | 10 | 1 | 0 | 1 | 0 | 0 | 1 | 0 |
| 80 | 10 | 0 | 0 | 0 | 1 | 0 | 0 | 0 |
| 81 | 11 | 1 | 1 | 0 | 0 | 0 | 1 | 0 |
| 82 | 11 | 1 | 0 | 1 | 1 | 1 | 1 | 0 |
| 83 | 11 | 0 | 0 | 0 | 1 | 0 | 1 | 1 |
| 84 | 11 | 0 | 1 | 1 | 1 | 0 | 0 | 0 |
| 85 | 11 | 1 | 1 | 0 | 1 | 1 | 0 | 1 |
| 86 | 11 | 1 | 0 | 1 | 0 | 0 | 0 | 1 |
| 87 | 11 | 0 | 0 | 0 | 0 | 1 | 0 | 0 |
| 88 | 11 | 0 | 1 | 1 | 0 | 1 | 1 | 1 |
| 89 | 12 | 0 | 1 | 1 | 0 | 1 | 1 | 0 |
| 90 | 12 | 0 | 1 | 1 | 1 | 0 | 0 | 1 |
| 91 | 12 | 1 | 0 | 1 | 0 | 0 | 0 | 0 |
| 92 | 12 | 0 | 0 | 0 | 1 | 0 | 1 | 0 |
| 93 | 12 | 1 | 1 | 0 | 0 | 0 | 1 | 1 |
| 94 | 12 | 1 | 0 | 1 | 1 | 1 | 1 | 1 |
| 95 | 12 | 1 | 1 | 0 | 1 | 1 | 0 | 0 |
| 96 | 12 | 0 | 0 | 0 | 0 | 1 | 0 | 1 |
| 97 | 13 | 0 | 1 | 1 | 1 | 1 | 1 | 0 |
| 98 | 13 | 1 | 1 | 0 | 0 | 1 | 0 | 0 |
| 99 | 13 | 1 | 0 | 1 | 1 | 0 | 0 | 0 |
| 100 | 13 | 0 | 0 | 0 | 1 | 1 | 0 | 1 |
| 101 | 13 | 0 | 1 | 1 | 0 | 0 | 0 | 1 |
| 102 | 13 | 1 | 0 | 1 | 0 | 1 | 1 | 1 |
| 103 | 13 | 0 | 0 | 0 | 0 | 0 | 1 | 0 |
| 104 | 13 | 1 | 1 | 0 | 1 | 0 | 1 | 1 |
| 105 | 14 | 1 | 1 | 0 | 0 | 1 | 0 | 1 |
| 106 | 14 | 1 | 0 | 1 | 0 | 1 | 1 | 0 |
| 107 | 14 | 1 | 0 | 1 | 1 | 0 | 0 | 1 |
| 108 | 14 | 1 | 1 | 0 | 1 | 0 | 1 | 0 |
| 109 | 14 | 0 | 1 | 1 | 0 | 0 | 0 | 0 |
| 110 | 14 | 0 | 1 | 1 | 1 | 1 | 1 | 1 |
| 111 | 14 | 0 | 0 | 0 | 1 | 1 | 0 | 0 |
| 112 | 14 | 0 | 0 | 0 | 0 | 0 | 1 | 1 |
| 113 | 15 | 0 | 0 | 0 | 0 | 0 | 0 | 0 |
| 114 | 15 | 0 | 1 | 1 | 0 | 0 | 1 | 1 |
| 115 | 15 | 1 | 1 | 0 | 1 | 0 | 0 | 1 |
| 116 | 15 | 0 | 0 | 0 | 1 | 1 | 1 | 1 |
| 117 | 15 | 1 | 1 | 0 | 0 | 1 | 1 | 0 |
| 118 | 15 | 1 | 0 | 1 | 0 | 1 | 0 | 1 |
| 119 | 15 | 1 | 0 | 1 | 1 | 0 | 1 | 0 |
| 120 | 15 | 0 | 1 | 1 | 1 | 1 | 0 | 0 |
| 121 | 16 | 0 | 1 | 1 | 1 | 1 | 0 | 1 |
| 122 | 16 | 0 | 1 | 1 | 0 | 0 | 1 | 0 |
| 123 | 16 | 1 | 0 | 1 | 1 | 0 | 1 | 1 |
| 124 | 16 | 1 | 1 | 0 | 1 | 0 | 0 | 0 |
| 125 | 16 | 0 | 0 | 0 | 0 | 0 | 0 | 1 |
| 126 | 16 | 1 | 0 | 1 | 0 | 1 | 0 | 0 |
| 127 | 16 | 0 | 0 | 0 | 1 | 1 | 1 | 0 |
| 128 | 16 | 1 | 1 | 0 | 0 | 1 | 1 | 1 |
